# Different rearing conditions alter gut microbiota composition and host physiology in Shaoxing ducks

**DOI:** 10.1038/s41598-018-25760-7

**Published:** 2018-05-09

**Authors:** Shumei Wang, Li Chen, Maozhang He, Junda Shen, Guoqin Li, Zhengrong Tao, Rurong Wu, Lizhi Lu

**Affiliations:** 1 Institute of Animal Husbandry and Veterinary Science, Zhejiang Academy of Agricultural Sciences, 310000 Hangzhou, China; 2 College of Chemistry and Life Sciences, Zhejiang Normal University, 321000 Jinhua, China; 3 State Key Laboratory of Pig Genetic Improvement and Production Technology, Jiangxi Agricultural University, 330045 Nanchang, China; 4 Lihong Poultry Industry Co., Ltd., 313000 Huzhou, China

## Abstract

Emerging evidences have linked the gut microbiota to poultry physiology. Gut microbiota composition in Shaoxing ducks were profiled under different rearing conditions: raised on the litter floor and the plastic mesh floor. A total of 46 and 39 luminal content samples from the duodenum, ileum, and cecum of the ducks reared under the two conditions were analyzed by 16S rRNA gene amplicon sequencing analysis. Proteobacteria (48.66%), Proteobacteria (33.38%), and Bacteroidetes (55.35%) were the dominant phyla in the duodenum, ileum, and cecum of the ducks reared on the litter floor respectively, while Firmicutes (30.80%), Firmicutes (66.62%) and Bacteroidetes (47.15%) were the topmost phyla in the duodenum, ileum, and cecum of the ducks reared on the plastic mesh floor. Physiologically, the height of villi and the ratio of villus height to crypt depth in the ileum and duodenum were significantly greater in the ducks reared on plastic mesh floor. Furthermore, our results demonstrate that the gut microbiota was significantly associated with the duck phenotypes, such as chest depth and serum estradiol levels (p < 0.05), which were altered by the different rearing conditions. Collectively, our results showed that the rearing floor types have an important effect on the gastrointestinal microbial composition of ducks.

## Introduction

The gut microbes of poultry and livestock play an essential role in the nutrient digestion and absorption, immune system development, and host protection against pathogens. The entire microbes found in a particular habitat, including bacteria, viruses, and funguses, is defined as microbiota^1,2^. In recent years, more and more studies focus on the gut microbes, which play an important role in our lives. Previous studies have demonstrated that diets^3,4^, breeding patterns, ages, and feed additives can impact the poultry gut microbes with respect to diversity, composition, and community structure^5,6^. Firmicutes, Bacteroidetes and Proteobacteria are the dominant phyla in poultry gut lumen. However, this bacterial community structure changes dynamically at different growth phases. Proteobacteria is dominant during the first three days of age, and Firmicutes starts to increase and dominate from day 4 until day 8 based on the study of Pekin ducks^5^. In contrast, Firmicutes dominates at all the ages of the broiler growth^7^. Recent studies showed that the compositions of phylum Bacteroidetes within the cecum of 12~14 week old Peking (*Anser platyrhynchos*) and Muscovy (*Cairina moschata*) ducks were similar to those of turkeys at an old age at 18 weeks^5,8^.

In addition to the impact of ages on the gut microbes, different regions of bowel also harbor different microflora. The duodenum is crucial for food digestion and absorption, has a lower pH than the hindgut, and is the region that absorbs most glucose and other nutrients within the small intestine^9–11^. Lactobacillus and Firmicutes account for the highest proportion of duodenum microbes. The cecum mainly decomposes carbohydrates and has a higher ability to absorb sugars actively at low concentrations compared with the jejunum. The dominant phyla of cecum microflora are Bacteroidetes and Firmicutes^12^. The small intestine, which consists of three different sections: the duodenum, jejunum, and ileum, is the portion of the digestive tract that connects the stomach and the large intestine. It contains small finger-like projections called villi, which increase the surface area of the intestine and is composed of specialized cells that transport different types of substances into the bloodstream. Although villi do not aid in the digestion of nutrients, they do help with nutrient absorption by increasing the surface area of the intestine. Together with the villi, another functional structure of the small intestine, known as the crypts, transports nutrient molecules from the digestive tract into the bloodstream, where they can be used by the body^13–15^. In addition, at the age of days 7 to 14, the chicken cecal contents resemble those of the chicken ileum. From day 14 forward, the compositions of the two regions become significantly different from each other^7^.

Avian intestinal microbiome is very different from that of monogastric mammals^16^. At present, most of the studies focus on the impact of feed additives on poultry, especially on the broilers. To date, very little is known about the gut microbiota composition of the Shaoxing ducks. In contrast, it is reported that over 24.5 million ducks are consumed in the United States each year (http://www.agmrc.org/commodities-products/livestock/poultry/ducks-and-geese/). This study aimed to investigate the changes of intestinal microbes in Shaoxing ducks under different rearing conditions and to explore the effects of intestinal microbes on improving the performance and immune function of Shaoxing ducks. A recent study revealed that the gut microbiota of barn raised ducks contained a significant population of Bacteroidetes in addition to Proteobacteria and Firmicutes at later developmental stages, though this phylum was absent in aviary raised ducks^5^. There are also studies showing that wild cranes had distinct compositions of gut microbiota from captive and artificially bred cranes. The greatest alpha diversity was found between captive cranes and wild cranes. A better understanding of the duck gut microbiomes could instruct the management practices, such as the use of prebiotics, probiotics, and enzymes in feeding practices, and help us understand the sources of pathogenic bacteria in diseased ducks. This study characterized the intestinal microbiome of Shaoxing ducks after the peak of egg production.

Shaoxing duck, a Chinese indigenous duck breed with excellent egg laying performance, is important in poultry farming of China. The experiment used the 16S rRNA sequencing approach to analyze the species and diversity of gut microbes and investigate the association of gut microbiota with the physiological and biochemical indexes of the ducks to help us better understand Shaoxing duck management practices. The data provide a reference for further studies of the Shaoxing ducks and may improve the duck husbandry to increase egg-yields and decrease incidence of diseases.

## Results

### Overview of microbiota composition of different gut regions under different rearing conditions in Shaoxing ducks

A total of 85 luminal content samples were used for the 16S rRNA gene sequencing (For details in Table 1). After quality control, we obtained an average of 38,731 high-quality tags for each sample. To avoid the effect of the sequencing depth on the measurement of microbial compositions, we rarefied the library size to 36,608 tags per sample using the rarefy function in QIIME pipeline^17^. With the 97% sequence similarity, we obtained an average of 690 OTUs for each sample. As a result, the dominant phyla in the ducks raised on the plastic mesh floor (RPMF) were the Firmicutes and Bacteroidetes. While Bacteroidetes is the most dominant phylum in the cecum (47% of sequences), Firmicutes is the dominant phylum in the duodenum and ileum. In contrast, the ducks raised on the litter floor (RLF) group also contained two major phyla, Proteobacteria and Bacteroidetes. Bacteroidetes was the most abundant phylum in the cecum, while Proteobacteria was dominant in the duodenum and ileum.

**Table 1:** Sample description of the Shaoxing ducks used in the study.

Gut region	Rearing condition	
	Reared on the litter floor (n = 39)	Reared on the plastic mesh floor (n = 46)
Duodenum	8	14
Ileum	15	16
Cecum	16	16

Luminal content samples were collected from the Duodenum, Ileum, and Cecum.

At the genus level, a total of 225 bacterial genera were identified based on the filtering criteria of each genus (more than 0.005% of total sequences)^18^. Across gastro-intestinal tract (GIT) regions, we found that the genera of *Bacteroides*, *Helicobacter* and *Prevotellaceae_Ga6A1_ group* were more abundant in the RLF group ducks, whereas in the RPMF group, *Romboutsia, Fusobacterium*, and *Brachyspira* were enriched. However, we observed a substantial GIT localization variation of the microbial composition. For instance, the average abundance of *Helicobacter* was 23.34%, ranging from 0.12% to 97.89% in the different GIT sections of the RPMF ducks, but in the RLF group, the average abundance of *Helicobacter* was 6.99%, ranging from 0.03% to 54.97% in the different GIT sections. The *Instestinibacter* abundance was 4.43%, ranging from 0 to 42.77% in the GIT sections of RPMF group, and 23.81% in the RLF group, ranging from 0.16% to 95.8% in the GIT sections.

### The differences of gastrointestinal tract microbial richness, diversity, and composition

**Figure 1:**
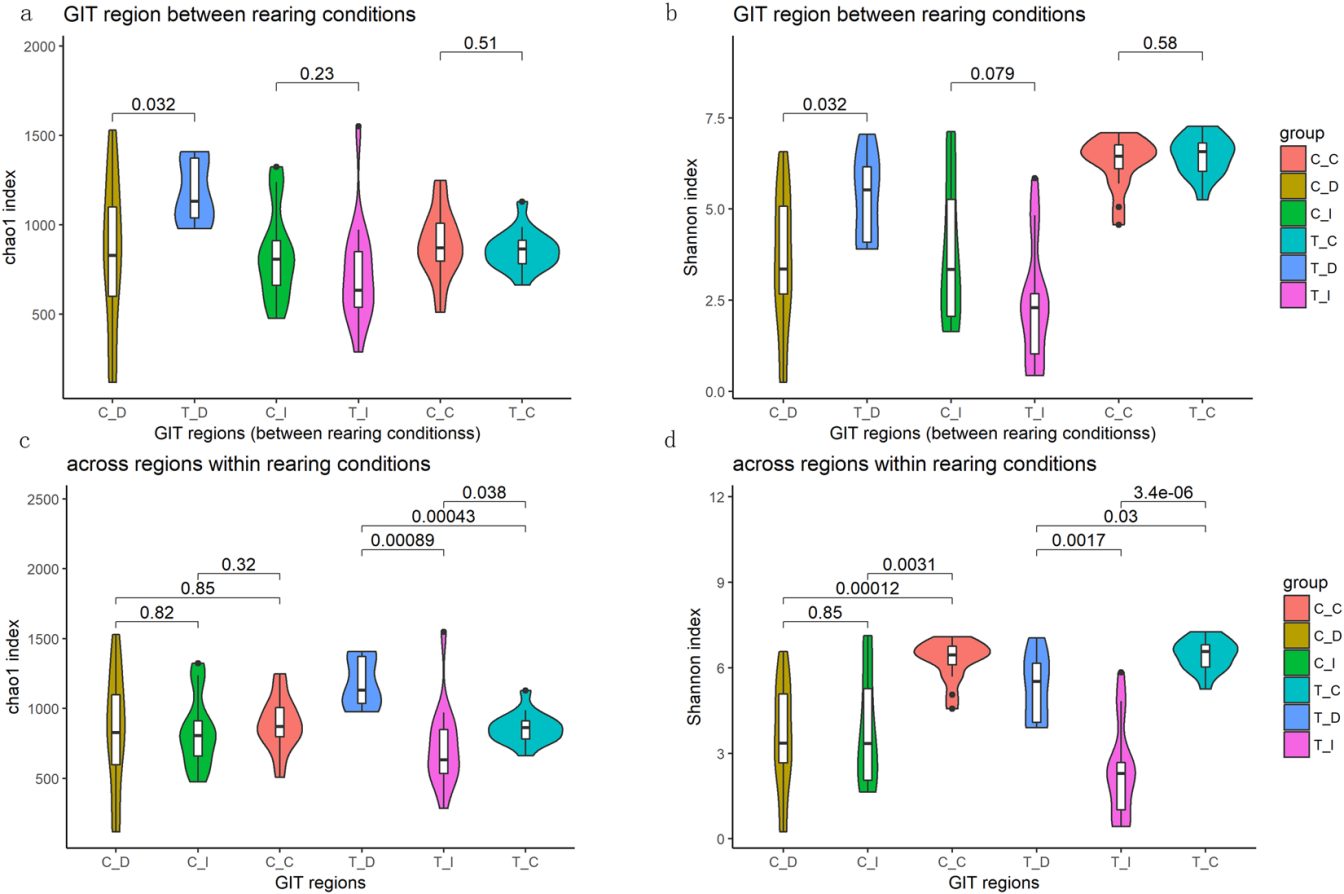
Alpha-diversity of luminal microbiota of the three gut regions under different rearing conditions. (**a**,**b**) Comparison of the richness (chao1 index) and diversity (Shannon index) of the specific GIT luminal microbiota under different rearing conditions. Violin plots showed that chao1 and Shannon indexes were significantly different between the 2 rearing conditions. (**c**,**d**) Comparison of the richness (chao1 index) and diversity (Shannon index) of the luminal microbiota of the three gut regions. Violin plots showed chao1 index were significantly higher in the duodenum of RPMF ducks. Shannon index were higher in the cecum independent of the rearing conditions. [^*^P < 0.05, ^**^P < 0.01, ^***^P < 0.001 for Student’s t-test].

Chao1 richness index and Shannon diversity index were used to evaluate the alpha diversity of GIT microbiota between the RLF and RPMF groups. In the duodenum, the chao1 and Shannon indexes were significantly higher in the RPMF group than the RLF group. In addition, there are no differences observed in ileum and cecum between the RPMF and RLF ducks (Fig. 1a,b).

**Figure 2:**
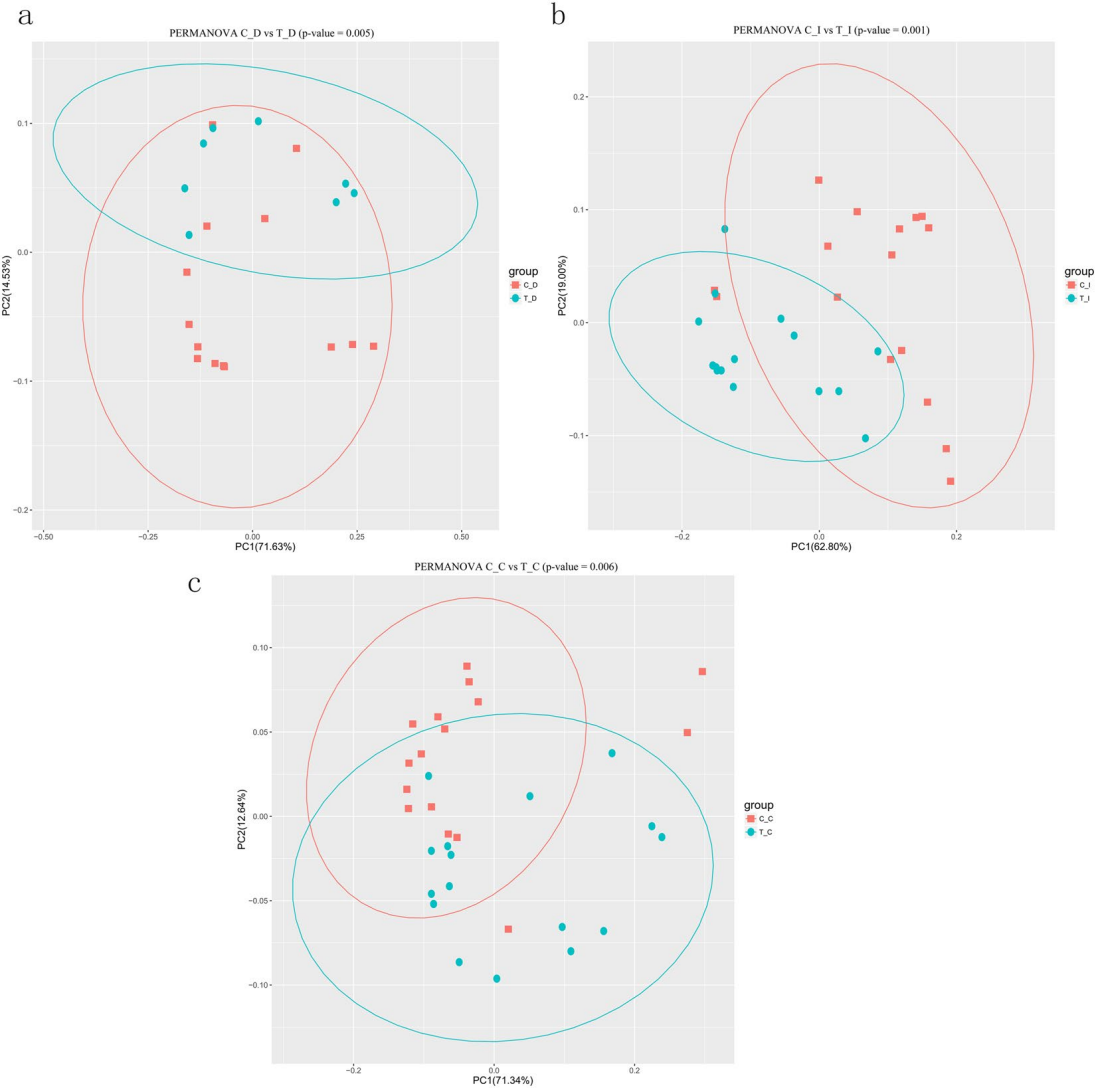
The overall luminal microbiota structures of Shaoxing ducks under different rearing conditions showed by Principal Coordinate Analysis of Weighted UniFrac Distances of RLF and RPMF ducks. Red and blue colors represent the RLF and RPMF duck samples, respectively. The significant separation between the 2 rearing conditions were observed in the duodenum (**a**), the ileum, (**b**), and the cecum (**c**).

Subsequently, the bacterial compositions of the three GIT regions under the 2 rearing conditions were compared based on the weighted UniFrac distance. Permutational multivariate analysis of covariance (PERMANOVA) was used to examine whether the matrix of major PCoA axes was dependent on the rearing conditions. According to the results of PCoA, the luminal microbial communities of the duodenum, ileum, and cecum were clustered tightly in the RPMF group but separated evidently in the RLF ducks (Fig. 2a,b,c).

To determine which bacterial taxa contributed most to the separation of the microbial communities. We first compared the same gut section under the two rearing conditions. At the phylum level, Firmicutes and Lentisphaerae were enriched in the duodenum of RPMF group, and Cyanobacteria were more abundant in the duodenum of RLF group. Meanwhile, we found the phyla Firmicutes and Fusobacteria were predominant in the ileum of the RPMF ducks, and two phyla, Proteobacteria and Cyanobacteria, were significantly increased in the ileum of RLF ducks compared with RPMF ducks. With respect to the cecum, we found that Elusimicrobia and Synergistetes were significantly increased in the RLF group, while the phylum Tenericutes was significantly reduced compared with the RPMF group (Supplementary Fig. 1A–C).

**Figure 3:**
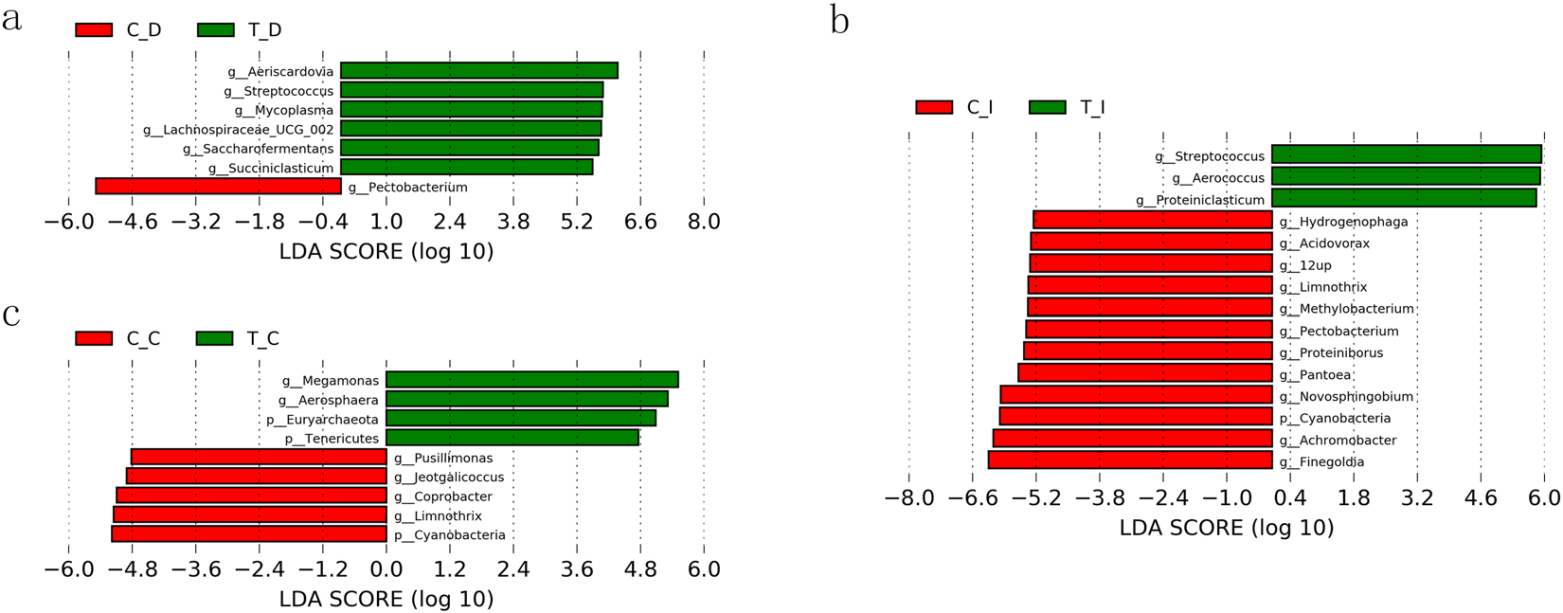
Histogram of the Liner discriminant analysis (LDA) coupled with effect size measurements identified the most differentially abundant genera between the duodenum (**a**), ileum (**b**), and cecum (**c**) of RLF and RPMF ducks. The RLF duck enriched genera are indicated with a negative LDA score, and the genera enriched in the RPMF ducks are indicated with a positive score. The criteria of the LDA score for discriminative features was >4.0, and the alpha value for the factorial Kruskal—Willis test among classes was <0.05.

At the genus level, Lefse results showed that 6 genera were found significantly higher in the duodenum of the RPMF group, and three genera were depleted compared to the RLF group (with the criteria of alpha value <0.05 and absolute LDA score > = 4). Furthermore, in the ileum, 12 genera were increased in the RLF ducks while 3 genera were enriched in RPMF ducks. At last, four genera were enriched significantly in the cecal samples of the RPMF group, while 5 genera were more abundant in the RLF group (Fig. 3a–c).

### The microbial community compositions of different GIT regions

In the RPMF ducks, the chao1 and Shannon index were significantly different among the duodenum, ileum, and cecum. While in the GIT of the RLF ducks, no significant difference of the chao1 index was found among the three GIT regions, but the Shannon index was found significantly different between the duodenum and cecum, as well as between the cecum and ileum (Fig. 1c,d).

**Figure 4:**
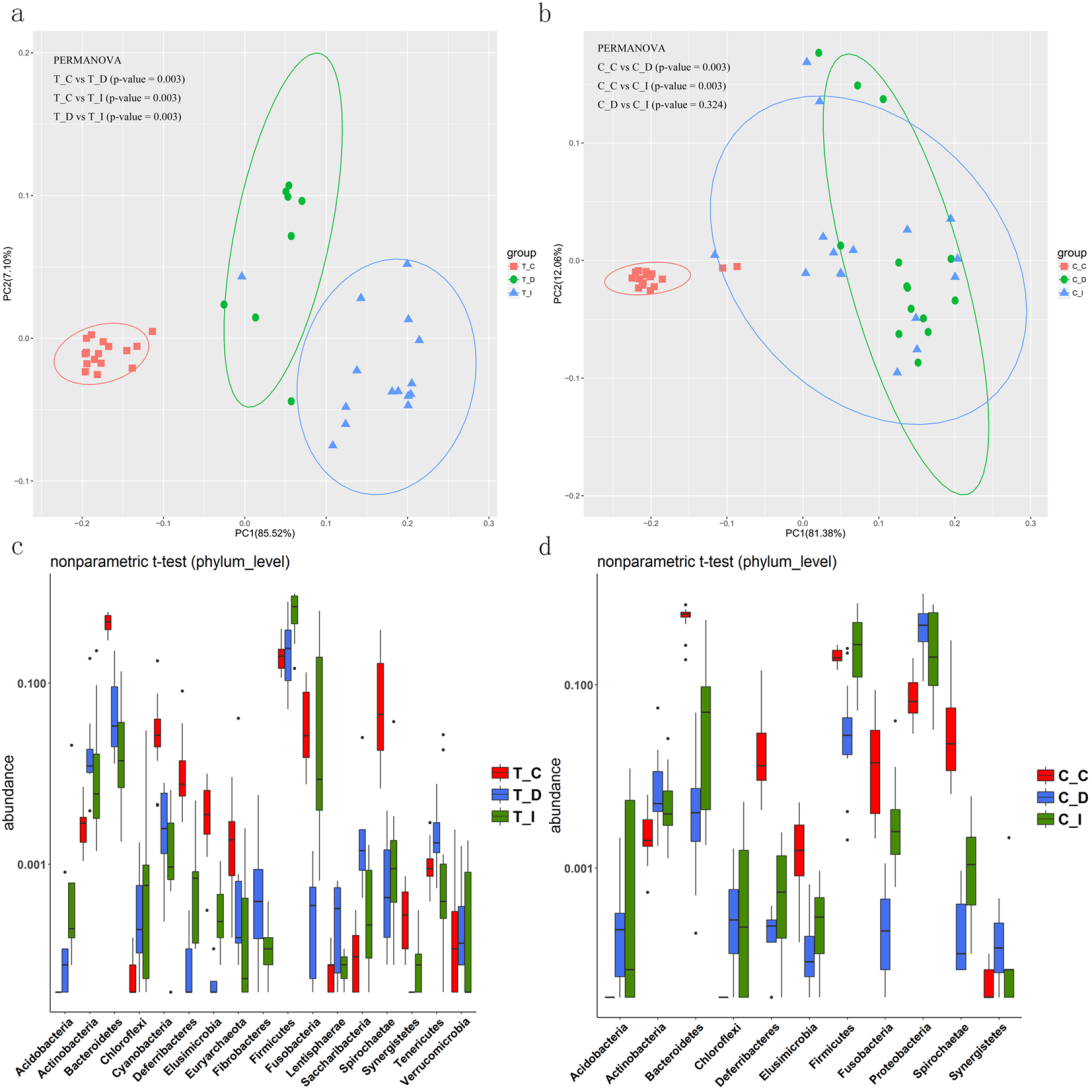
The overall luminal microbiota structures and compositions of three gut regions of Shaoxing ducks under the same rearing condition. (**a**,**b**) Principal Coordinate Analysis of Weighted UniFrac Distances of the duodenum, ileum, and cecum luminal samples under each rearing condition. (**c**,**d**) Comparison of luminal microbiota compositions of the three gut regions at the phylum level under the 2 rearing conditions was performed by Nonparametric Wilcox t-test analysis.

Besides, we compared the bacterial communities in the duodenum, ileum, and cecum under the same rearing condition. In the RPMF ducks, the structures of microbial communities in three intestinal sections displayed a significant separation among each other with the duodenum in the middle of the ileum and cecum samples (Fig. 4a). In the RLF ducks, the principal coordinate analysis (PCoA) based on weighted UniFrac distance showed that the cecal microbial communities were significantly separated from the ileal and duodenal sections, but the microbial communities of the duodenum and ileum were overlapped (Fig. 4b).

When bacterial compositions were compared regionally under the same rearing conditions, we observed the phyla Actinobacteria, Lentisphaerae, Tenericutes, and Saccharibacteria significantly dominated the duodenum of the RPMF group. Firmicutes and Acidobacteria were predominant in the ileum, while Spirochaetae and Bacteroidetes were more enriched in the cecum (Fig. 4c). Within the RLF ducks, Actinobacteria, Proteobacteria, and Synergistetes were significantly enriched in the duodenum. Notably, we found the Firmicutes became predominant in the ileum. Similarly, Bacteroidetes and Spirochaetae had an increased abundance in the cecum (Fig. 4d).

At the genus level, 5 genera were significantly increased in the ileal samples compared with the other 2 sections of GIT, and 24 and 35 genera were enriched in the cecal and duodenum samples in the RPMF ducks, respectively (Supplementary Fig. 2A). On the contrary, we observed 4 genera were more enriched in the ileum, 6 genera were significantly increased in the duodenum samples, and 29 genera were abundant in the cecal luminal samples in the RLF ducks (Supplementary Fig. 2B).

### Predict molecular functions of the luminal microbiomes under the two rearing conditions

**Figure 5:**
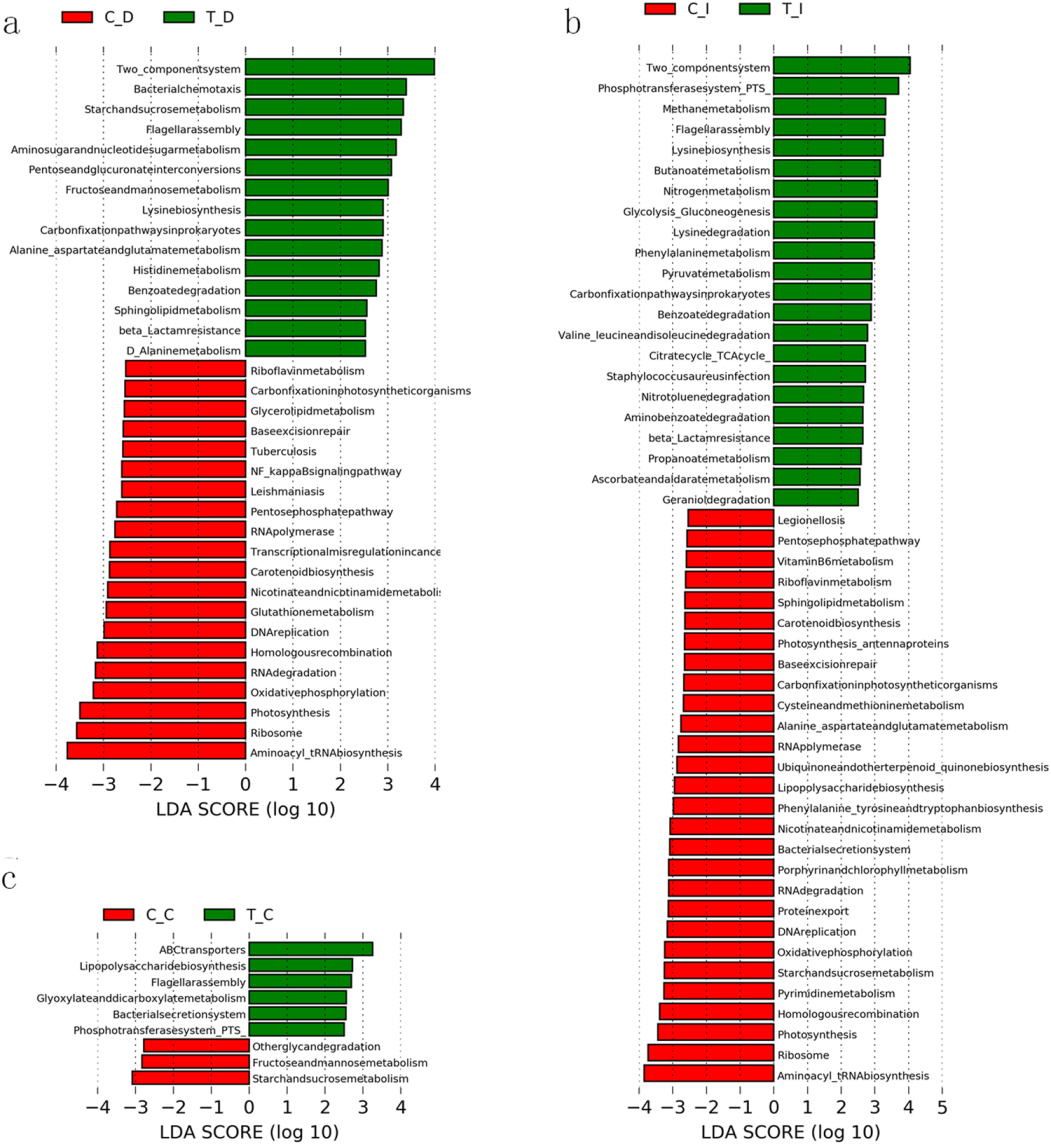
The different KEGG functional pathways between the ducks under the two rearing conditions. Linear discriminate analysis (LDA) coupled with effect size measurements identified the most differentially abundant functional genes between the duodenum (**a**), ileum (**b**), and cecum (**c**) samples of the RLF and RPMF ducks.

The GIT region-associated microbiomes under each rearing condition were further analyzed with PICRUS to predicted their potential functions and gain insight of their role in management practices. When compared the RPMF with the RLF ducks, the pathways of two-component system, bacterial chemotaxis, starch and sucrose metabolism, and flagellar assembly were found increased in the duodenum of the RPMF group (Fig. 5a). In the ileal sample, we demonstrated the functional switch mainly included significantly increased representation of two-component system, phosphotransferase system (PTS), flagellar assembly, butanoate metabolism in the RPMF ducks (Fig. 5b). In the cecum, we identified a total of 9 significant different KEGG pathways between the RPMF and RLF samples, among which ABC transporters, flagellar assembly, bacterial secretion system, and PTS were significantly increased in the RPMF group. However, we found the starch and sucrose metabolism and fructose and mannose metabolism pathways were enriched in the RLF group (Fig. 5c).

Furthermore, we compared the luminal bacterial functional KEGG pathways across the gut regions under the same rearing conditions. Of the 17 predicted functional pathways that showed significant differences across the intestinal regions in the RPMF group, PTS, and flagellar assembly were increased in the ileac samples compared with the duodenum and cecum. Besides, we observed a total of 88 pathways had significantly different abundance among the duodenum, ileum, and cecum. For example, the pathways of ABC transporters, PTS, Flagellar assembly, and butanoate metabolism were relatively more abundant in the ileum than the other 2 regions (Supplementary Fig. 3A). Furthermore, we observed 10 predicted KEGG genes were significantly enriched in the duodenum, and 2 pathways, PTS and flagellar assembly, were more abundant in the ileum. At last, 5 KEGG pathways were predominant in the cecal microbiome (Supplementary Fig. 3B).

### Morphological description of intestinal and liver paraffin section

It is well documented that the structure and morphology of villi play a substantial role in the digestion and absorption of nutrients in the GIT^19^. In the current study, the histomorphological analysis of the intestine and liver were performed by hematoxylin-eosin (HE) staining of paraffin sections. The morphology of the GIT and liver were shown in Supplementary Fig. 4A–F and Supplementary Table 2. We observed a diverse morphology among the three GIT regions, as well as under the two different rearing conditions. The ileal paraffin sections showed an increased height of villi compared with the duodenum and cecum, which confers a greater villi surface area to the ileum. In addition, the ratios of VH/CD in the duodenum and ileum were both significantly higher in the RPMF group than the RLF group (P = 0.014 and 1.0E-5, respectively), though the crypt depth of the duodenum and ileum in the RPMF group were significantly decreased compared with the RLF group. These morphological changes inferred that the ducks raised on the plastic mesh floor had a pronounced improvement in the digestive and absorptive functions of the small intestine. Nonetheless, the ileal mucosal thickness (MT) of the RLF group was found significantly increased compared with that of the RPMF group (Supplementary Table 1).

The livers from the RPMF group of ducks have the same histological characteristics as those of the RLF group (Supplementary Fig. 4G,H). Histological examination showed the ducks under both rearing conditions had some degree of inflammation, which was in accordance with the anatomical observation of the livers.

### Changes in liver and serum biochemical indexes under different rearing conditions

The effects of different rearing conditions on the duck liver and serum biochemical indexes were determined. The liver malondialdehyde (MDA) level and lipopolysaccharide (LPS) level were significantly increased in the ducks reared on the plastic mesh floor. The superoxide dismutase (SOD) and glutamic-oxalacetic transaminase (AST) activity were significantly lower in the ducks reared on the plastic mesh floor than in the ducks reared on the litter floor. However, there was no significant difference in the activity of liver total antioxidant capacity (T-AOC), alanine transaminase (ALT), lactic dehydrogenase (LDH), and tumor necrosis factor-α (TNF-α) between the 2 groups of ducks (Supplementary Table 2).

The data of serum biochemical indexes were shown in Supplementary Table 3. We found the levels of Interleukin-4 (IL-4) and estradiol-E2 (E2) were both significantly increased in the RPMF group of ducks compared to the RLF group. Besides, no significant difference was observed in other serum indexes.

### Growth and body size traits under different rearing conditions

Duck growth and body size traits (Supplementary Table 4) were measured, and the correlations between the traits and rearing conditions were analyzed. Only the chest width (CW) of the RPMF group showed a significant increase compared to the RLF ducks. There was no significant difference in others growth and body size traits.

### Phenotypes associated with cecal microbiota composition under RPMF and RLF conditions

The cecum has been reported as a place with a lower pH and a higher content of easily fermentable compounds than the more distal regions of the GIT. Consequently, it harbors very different microbial communities from the rest intestine. Hence, we next performed the multivariable association analysis of each phenotypic parameter and the 347 unique OTUs (shared in 50% of individuals) in the cecum. The significantly different traits between the two rearing conditions were selected, including superoxide dismutase, total antioxidant capacity, malondialdehyde, lipopolysaccharide, glutamic-oxalacetic transaminase, IL-4, estradiol, and chest depth. Finally, we identified a total of 29 associations between 6 traits and 21 unique bacterial OTUs at a p-value < = 0.05 in the RLF group: 4, 9, 5, 3, 7, and 1 OTUs were significantly associated with the AST, estradiol, IL-4, LPS, SOD, and MDA levels in the Shaoxing ducks, respectively. In the RPMF group, 40 associations between 6 traits and 22 unique bacterial OTUs were identified: 5, 9, 6, 6, 5, and 8 OTUs were significantly associated with the chest depth, AST, estradiol, IL-4, MDA, and SOD levels, respectively.

**Figure 6:**
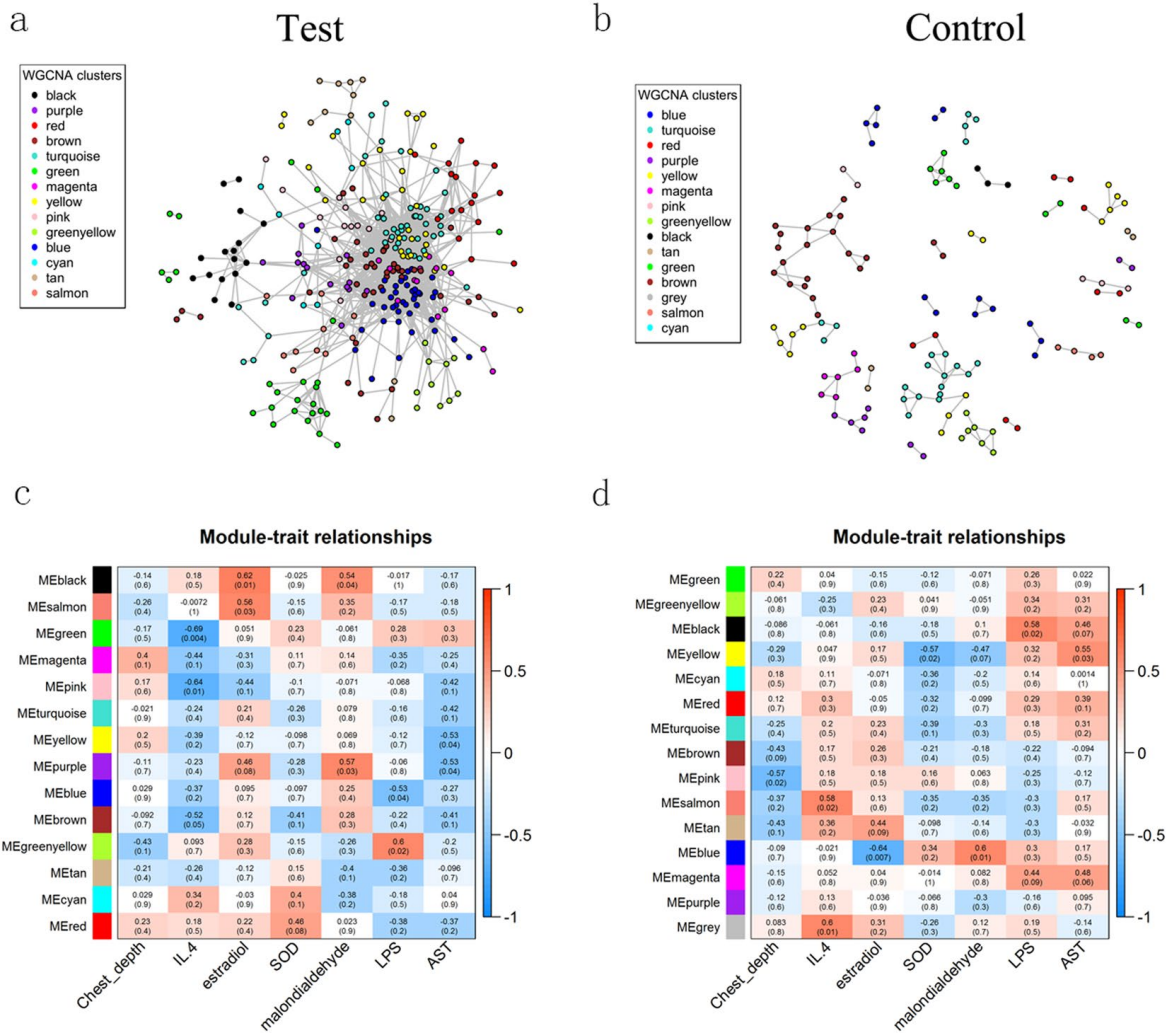
Construction of OTU co-occurrence network and module-trait associations of cecal microbiota of the RLF and RPMF ducks. (**a**,**b**) OUT co-occurrence network was constructed. OTU (*nodes*) are represented according to WGCNA module colors. (**c**,**d**) Module-trait associations are shown. Each cell of the matrix contains the correlation between one OTU module and a trait and the corresponding *p* value. Red and blue colors represent positive and negative correlations, respectively. *IL.4*, Interleukin 4; *SOD*, Superoxide dismutase; *LPS*, Lipopolysaccharide; *AST*, Aspartate transaminase.

However, the poultry gut is a complex and dynamic ecosystem, which consists of a diverse microbiota with a variety of functional relationships. For the aim of detecting the relationships between different strains of the gut microbiome and their potential effect on the host physiology and body size traits, we constructed a network of co-occurrence OTUs and interrogated the network for modules using weighted gene co-expression network analysis (WGCNA) (Fig. 6a,b).

The module-traits associations confirmed several previously detected traits-OTUs associations. For example, the blue module in the RLF group, which contains the OTUs from genus *Epulopiscium*, was negatively correlated with the estradiol level. Similarly, the salmon module in the RPMF group, which contains the OTUs from family *Ruminococcaceae*, was positively correlated with the estradiol level. These associations were also observed with the single OTU tests. Nonetheless, we also detected some novel OTUs within the modules that were significantly associated with the traits, which provided further insights into gut microbiome-traits relationships (Fig. 6c,d).

## Discussion

It has been well recognized that the microbial localization, diversity, and composition varied across the gastrointestinal tract and are regulated by driven factors, such as PH, oxygen level, *et al*. Here, we present a comparative gut microbiota structural analysis of the ducks reared under different rearing conditions. We also conducted a comprehensive association study of the rearing conditions, as well as the gut regions, with the gut microbiome compositions and functions. At last, we explored the associations of GIT morphological changes, liver and serum biochemical indexes, and growth and production performance with the rearing conditions.

The findings of the present study showed a remarkable shift of the alpha diversity of bacteria between the GIT segments of the two group ducks as indicated by the chao1 values and Shannon index, which is in accordance with previous studies in the pig and other livestock^20,21^. Furthermore, our results demonstrated the representative taxonomic groups within the duck GIT were Firmicutes, Bacteroidetes, and Proteobacteria. However, the abundance and composition of genera varied considerably among the GIT regions. These results confirmed the previous reports, which showed that the differences in the luminal PH values of different regions, as well as nutrient supplies, gut motility, and host secretions, would impact gut microbiota community structures^22–26^. In general, we investigated three GIT regions (the duodenum, ileum, and cecum) of the ducks reared on either the plastic mesh floor or the litter floor, which allowed us to determine whether different rearing conditions can result in changes of the GIT microbiome community and functions.

Intriguingly, our taxonomic classifications showed Firmicutes and Bacteroidetes were the most abundant bacteria in the cecum, which is in concert with previous studies of other animals^20^. We observed that genera *Megamonas* and *Aerosphaera* were significantly enriched in the RPMF ducks. Previous reports showed that *Megamonas* were mainly present in the cecal microbiota of adult hens, which is in line with our results that the cecum harbors a relatively high abundance of *Megamonas* than the duodenum and ileum. Furthermore, Polansky *et al*.^27^ demonstrated *Megamonas* was one of the major propionate producers in the phylum Firmicutes and could encode enzymes involved in melibiose and alanine metabolism. In contrast, genera *Coprobacter*, *Jeotgalicoccus*, and *Pusillimonas* were more abundant in the RLF ducks, and *Coprobacter* was described as a propionic and acetic acids producer^28^. However, the metabolic function prediction of the RPMF cecal microbes showed an increase of ABC transporters, phosphotransferase system transporters, and flagellar assembly pathways, which are indicators of bacterial uptake of carbohydrates, glycerol, and phosphate by the gut microbiome. The function prediction of the RPMF cecal microbes also showed the decreased capabilities of starch and sucrose metabolism and fructose and mannose metabolism compared with the RLF ducks. These observations suggest that the ducks reared on the plastic mesh floor enables its microbial assemblages within the cecum to increase their capabilities for butyrate production and nutrient absorption.

In the ileum, Firmicutes and Proteobacteria were the top two phyla in the RLF ducks, whereas Firmicutes and Fusobacteria were the major phyla in the RPMF ducks. The over-representative genera in the RPMF ducks are *Streptococcus*, *Aerococcus*, and *Proteiniclasticum*. In addition, the genera predominated in the RLP ducks were *Finegoldia*, *Achromobacter*, *Pantoea*, and *Proteiniborus*. The majority of the genera observed in the RLP ducks were opportunistic pathogens. Several previous findings supported that these genera of microbes caused the diseases of human and animals with the exception of the short-fatty acids producer *Proteiniclasticum* and lactic acid producer *Streptococcus* that were found enriched in the RPMF group^29^. This transition coincided with the observation that the predicted functional genes involved in the valine-leucine and isoleucine degradation and propanoate metabolism were more abundant in the RPMF ducks. The great proportion of these genes reflected the increase of functions affiliated with amino acid metabolism, implying that the amino acid degradation is more necessary for the ileal microbiota in the RPMF ducks. In addition, we speculated that the over-representation of pathogens that were found in all the ducks independent of the rearing conditions might be affected by age. However, the more prevalent pathogens in the RLF ducks suggested the impact of different rearing conditions.

Due to the proximal position to the stomach, which confers the duodenum the low PH and rapid passage of intestinal contents, the duodenum had low bacterial counts. Firmicutes and Proteobacteria were the major microbial groups in the RPMF ducks, whereas the phyla Proteobacteria and Cyanobacteria were the top two phyla in the RLF group. With a fine taxonomic classification, we detected the microbes whose abundance was significantly altered between the RPMF and RLF ducks. The genera with higher abundance in the RPMF ducks than the RLF ducks included *Streptococcus*, *Lachnospiraceae_UCG_002*, *Saccharofermentans*, and *Succiniclasticum*. However, *Pectobacterium* was more abundant in the RLF ducks. *Streptococcus* has been reported to be a normal component of GIT microflora. *Streptococcus* and *Saccharofermentans* ferment carbohydrates or starch to produce acetate, lactate, fumarate, and hydrogen peroxide as the end products. Furthermore, lactic acid and short-chain fatty acid could reduce the PH of the gut and then inhibits the growth of other bacteria, including the enteropathogens^30,31^. These results were in line with the predicted molecular functions of the microbiota in the RPMF ducks: the glycometabolism pathways, including starch and sucrose metabolism and fructose and mannose metabolism, were more abundant in the RPMF ducks. In addition, the enrichment in two-component system and Flagellar assembly also benefited the energy absorption.

Furthermore, we investigated the associations of cecal microbiota with the rearing condition-related traits. The gut microbiome is a complex and metabolically active community, which can produce a large number of metabolites influencing host phenotypes. In the present study, several new findings were presented. First, we identified a number of associations between cecal microbiota and AST, chest depth, estradiol, IL_4, LPS, MDA, and SOD under the two different rearing conditions. Second, we found a group of co-occurrence microbes and demonstrated the single OTU-based module-trait associations, as well as provide some new insights into microbiota-trait relationships. The predictable differences in the microbes-traits association results between the ducks raised in the two conditions might be explained by the different exposure degrees to the ground and feces of the two duck groups. The RLF ducks were allowed to play in the pond, which gave the ducks more exposure to the pathogenic bacteria and potentially increased the community transmission of the pathogenic bacteria. As a result, more opportunistic pathogens were found in the gut microbiota of the RLF ducks, and the ascites were more common in the RLF ducks than the RPMF ducks.

In summary, Shannon index showed that the gut microbiota of the cecum was much more diverse and independent of rearing conditions, which was in concert with the previous results in broiler chickens^32–34^. However, we found the chao1 index was highest in the duodenum regardless of rearing conditions. We speculate that it was caused by the proximal position of the duodenum to the esophagus, and the microbes from feed increased its bacterial richness. In the present study, the microbiota was found to separate evidently in different intestinal regions and under different rearing environments. A number of previous studies had the similar results. For instance, Cui *et al*.^35^ found a clear separation of microbial compositions between the free range and caged hens, as well as across different GIT sections. Looft *et al*.^36^ showed that the ileum, cecum, and colon have distinct microbial communities in swine at taxonomic levels. Collectively, our results revealed that the different GIT regions of Shaoxing ducks have different microbial community compositions, but also contain shared microbiota. We speculated that these distinctions between ducks and other animals were likely shaped by different physicochemical conditions and specified nutrient requirements^37^. Particularly, the taxonomic switch between the RPMF and RLF ducks support the assumption that rearing conditions might play an important role in duck GIT microbial community structures. Most of the short-chain fatty acid production strains, as well as the nutrient absorption genes, were found more abundant in the RPMF ducks. In addition, the RPMF ducks possess a better intestinal morphology, blood physiological and biochemical indexes, and growth traits. This study suggested that rearing the ducks on the plastic mesh floor have a significantly beneficial effect on the microflora composition and function of Shaoxing ducks, and may promote the host physiological status, intestinal health, and duck performance.

## Methods

### Animal Feeding and Management

The study was conducted in a commercial aviary at Lihong Poultry Industry Co., Ltd. Sixty-four Shaoxing ducks at 300 days of age were selected randomly from a total of one thousand and two hundred ducks that were reared on the plastic mesh floor (the RPMF group), and another 64 Shaoxing ducks were selected from two thousand ducks reared on the litter floor (the RLF group). These ducks were moved to the new pens and continuously kept under RPMF and RLF conditions for another 100 days. The ducks were fed ad libitum with the same commercial formula diet, which mainly contained corn, soya bean meal, rapeseed dregs, and wheat-middlings. The RPMF group was managed on the plastic mesh floor over the ground to keep the ducks away from dirt, and the RLF group was reared on the ground with a fence. The flock density was standardized across the two pens based on the industry standard (~0.17 m^2^/duck). All the experimental ducks were healthy and did not receive any antibiotic treatments during the period of experiment. The luminal content samples were obtained at the end of the experiment.

### Ethic statement

The animal care and use protocol in this study was reviewed and approved by the Research Ethics Committee and the Animal Ethical Committee of the Zhejiang Academy of Agricultural Sciences and Zhejiang Normal University. All methods used in this study were performed in accordance with protocols approved by the Laboratory Animal Management Committee of the Zhejiang Academy of Agricultural Sciences and the Ministry of Science and Technology of the People’s Republic of China.

### Phenotypic Measurement and Luminal content Collection

A total of 16 ducks were selected randomly in each group and sacrificed by cervical dislocation after 8-hour fasting, the following body traits were measured with a vernier caliper and a measuring tape: body weight, body slanting length, keel length, chest width, chest depth, pelvic width, tibia length, shank girth, and semi-submersible length. The luminal contents of the duodenum, ileum, and cecum were collected with 2-ml sterile plastic cryo tubes and immersed in liquid nitrogen immediately. After transported to the laboratory, the samples were stored at −80 °C until used. T-D, T-I, and T-C were used to label the duodenum, ileum, and cecum samples from the RPMF group, respectively, while C-D, C-I, and C-C represented the duodenum, ileum, and cecum samples from the RLF group. Next, the gut tissue samples of the duodenum, ileum, and cecum with an approximate length of 2-cm were resected from the middle portion of each segment. The above samples, as well as the liver samples, were soaked into 4% paraformaldehyde after rinsed with sterilized phosphate buffered solution (PBS) buffer, then embedded in paraffin for hematoxylin and eosin (H&E) staining. Microscopic images (AxioObser Z1, Germany) were captured at the magnifications of ×50 and ×100. For every section of the intestine, eight different fields of view per section of ten consecutive sections were used. The villus height (VH, from the apical to the basal villi), which corresponded to the superior portion of the crypts, the crypt depth (CD, from the base to the transition region between the crypt and villus), and the ratio of VH/CD were determined using the images^38^.

### Measurement of Liver and Blood Physiological-Biochemical Indexes

Blood samples and liver tissue were collected from 16 ducks of each group. After slaughter, approximate 5 ml of blood was collected into non-anticoagulant blood tubes from the carotid artery of each duck and centrifuged at 3,000 × g at 4 °C for 10 min. The supernatant was transferred to a new sterile centrifuge tube, frozen immediately at −80 °C, and shipped on dry ice. A series of serum indexes were measured, including the concentrations of lysozyme, interleukin 2 (IL-2), interleukin 4 (IL-4), adrenaline and estradiol, etc. Liver tissue was homogenized and centrifuged at 3,000 × g at 4 °C. The supernatant was used to measure the levels of Superoxide dismutase (SOD), total antioxidant capacity (T-AOC), malondialdehyde (MDA), lipopolysaccharide (LPS), alanine transaminase (ALT), glutamic-oxalacetic transaminase (AST), lactic dehydrogenase (LDH), and tumor necrosis factor-α (TNF-α). The levels of above factors were determined with Enzyme-Linked ImmunoSorbent Assay and Biochemical Assay using commercial kits.

### Bacterial DNA extraction and Sequencing of 16S rRNA

Luminal content DNA was extracted using QIAamp DNA Stool Mini Kit (Qiagen, Germany) according to the manufacturer’s protocol^39^. The concentration and integrity of DNA were measured by the NanoVue-108493 and 1.5% agarose gel electrophoresis. The DNA samples were stored at −80 °C until processed for amplification. To construct 16S rDNA sequencing libraries, the V3-V4 regions of the 16S rDNA gene was amplified from the DNA samples by PCR using primer set of 341 F (5′-CCTAYGGGRBGCASCAG-3′) and 806 R (5′- GGACTACNNGGGTATCTAAT-3′). The PCR mixture (30 μL) contained 15 μL of Phusion Master Mix (2X), 10 ng Genomic DNA, 3 μL of Bar-PCR primer F (2 μM), and 3 μL of Primer R (2 μM). PCR was performed using the following cycles conditions: an initial denaturation step at 98 °C for 1 min, followed by 30 cycles of 98 °C for 10 s, 50 °C for 30 s, 72 °C for 30 s, and a final extension step at 72 °C for 5 min. The products were visualized by electrophoresis on 1% agarose gel, purified by the GeneJET™ Gel Extraction Kit (Thermo scientific-fermentas, Shanghai, China). Paired-end libraries were constructed using the NEBNext® Ultra™ DNA Library Prep kit for Illumina. The library quality was assessed on the Qubit and Agilent Bioanalyzer 2100 system. At last, the libraries were sequenced on an Illumina HiSeq. 2500 platform, and 250 bp paired-end reads were generated.

### Read Assembly and Taxonomic Classification

Paired-end clean reads were assigned to the samples based on their unique barcodes, trimmed by cutting off the barcode and primer sequences, and then merged using FLASH (V1.2.11)^40^. Quality filtering of the raw tags was performed under specific filtering conditions according to the QIIME to obtain the high-quality clean tags^41^. The tags were compared with the reference database (Gold Database) using UCHIME algorithm^42^ to detect chimera sequences, which were removed. The resulted effective tags were analyzed with Uparse software (V8.1.1861)^42^. Sequences with ≥97% similarity were assigned to the same operational taxonomic units (OTUs). Representative sequences for each OTU were screened for further annotation. For each representative sequence, the GreenGene Database^43^ was used to annotate taxonomic information based on RDPclassifier algorithm (Version 11.4)^44^. In order to study the phylogenetic relationship of different OTUs and the difference of the dominant species in different samples (groups), multiple sequence alignments were conducted using the MUSCLE software (Version 3.8.31)^45^. OTU abundance information was normalized using a standard of sequence numbers corresponding to the sample with the least sequences. Subsequent analyses of alpha diversity and beta diversity were all based on the normalized output data.

### Bacterial Diversity Analysis

Alpha diversity was applied to analyze the species diversity complexity of a sample through 6 indexes: Observed-species, Chao1, Shannon, Simpson, ACE, Good-coverage. All these indexes in our samples were calculated with QIIME and displayed with R software (Version 3.2.2). Beta diversity analysis was used to evaluate the differences in species complexity of samples. Beta diversity on both weighted and unweighted UniFrac was calculated by QIIME software. Cluster analysis was preceded by principal Coordinate analysis (PCoA), which was applied to reduce the dimension of the original variables using the FactoMineR package and ggplot2 package in R software (Version 3.2.2).

### Co-occurrence network analysis

Weighted gene co-expression network analysis (WGCNA)^46^ was used to generate a co-occurrence network based on the OTU log10-transformed count data. Briefly, we considered a signed network and chose the minimal beta value satisfying the scale free topology criteria (optimal beta equal to 5 in RPMF group and 8 in RLF group). We set up values 2 and 10 as the parameters of deepSplit and minModuleSize for the dynamic tree cut function^47^. The module eigenOTU, which was defined as the first principle component of a module, was used to calculate the Pearson correlation between a module and a metabolic trait. The significance of the correlation was determined by a Student asymptotic p-value. Visualization of the network was performed using the gplot function available in the sna R package.

For co-occurrence network comparisons, we constructed networks of the cecum under different rearing conditions.

### 16 S rRNA gene-based prediction of metagenomic functions

To evaluate the functional capabilities of gut microbiome of each luminal section and each rearing conditions, PICRUSt^48^ was used to predict the functions of microbial communities. In addition, differentially abundant functional components across different intestinal sections and under different rearing conditions were analyzed using LEfse algorithm^49^. Differences with liner discrimination analysis scores >2.5 and p-value < 0.05 were considered statistically significant.

### Statistical analysis

To test the microbial community compositional differences, two-tailed nonparametric Mann-Whitney U tests were performed to compare the variables of the two groups with non-normally distributed data. Multiple group compositions were compared using Kruskal-Wallis tests. We used analysis of variance of permutation (PERMANOVA) from R’s package vegan to compare the effect of rearing conditions on microbial community structures. A p-value < 0.05 was taken as statistical significance.

In addition, to test the association of each taxonomy and species, raw OTUs table was filtered to remove low abundant OTUs, and finally, 347 OTUs (shared in more than 50% of samples) were selected. The abundance of each species was logarithm 10 (log10) transformed. The association of an individual species with each carcass trait was assessed using boosted additive generalized linear models available in the MaAsLin R package with 5% significance level after multiple testing corrections. MaAsLin^50^ is a multivariate statistical framework that finds associations between clinical metadata or microbial community abundance and functions. In our analysis, we used the default settings of MaAsLin.

### Availability of materials and data

The datasets generated during and/or analysed during the current study are available from the corresponding author on reasonable request.

## Electronic supplementary material

Supplementary information
