# Supplementary material for: Different rearing conditions alter gut microbiota composition and host physiology in Shaoxing ducks
Source: Sci Rep. 2018 May 9;8:7387. doi: 10.1038/s41598-018-25760-7 (PMC5943461; doi:10.1038/s41598-018-25760-7)
Supplement: Supplementary file 1 — Supplementary information [file 41598_2018_25760_MOESM1_ESM.pdf]

Different rearing conditions alter gut microbiota composition and host physiology in Shaoxing ducks

Shumei Wang[1,2], Li Chen[1], Maozhang He[3], Junda Shen[1], Guoqin Li[1], Zhengrong Tao[1], Rurong Wu[4], Lizhi Lu[1*]

*To whom correspondence should be addressed

Address for correspondence

Lizhi Lu (lulizhibox@163. com)

Institute of Animal Husbandry and Veterinary Science, Zhejiang Academy of Agricultural Sciences

Hangzhou, 310000, P.R China

Phone: 0086-133-06813018

Fax: 0086-133-06813018

Supplementary Figure 1 Differential luminal microbiota of the duodenum (A), ileum (B), and cecum (C) at the phylum level between the two rearing conditions detected by STAMP software.

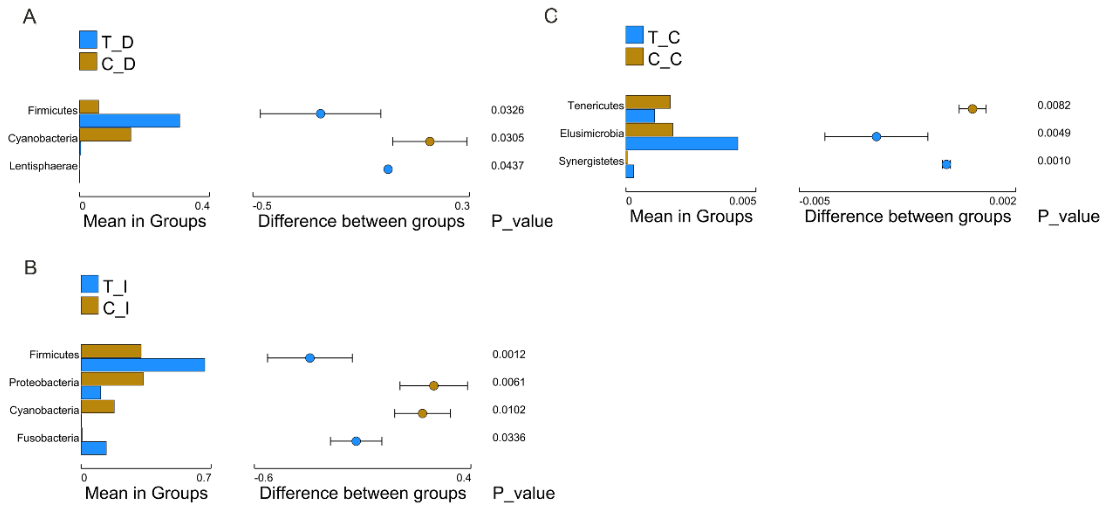

Supplementary Figure 2 Histogram of the Liner discriminant analysis (LDA) coupled with effects size measurements identified the most differentially abundant genera of 3 gut regions under the two rearing conditions. (A) The significant differential genera of the 3 gut regions of the RPMF ducks. (B) The significant differential genera of the 3 gut regions of RLP ducks.

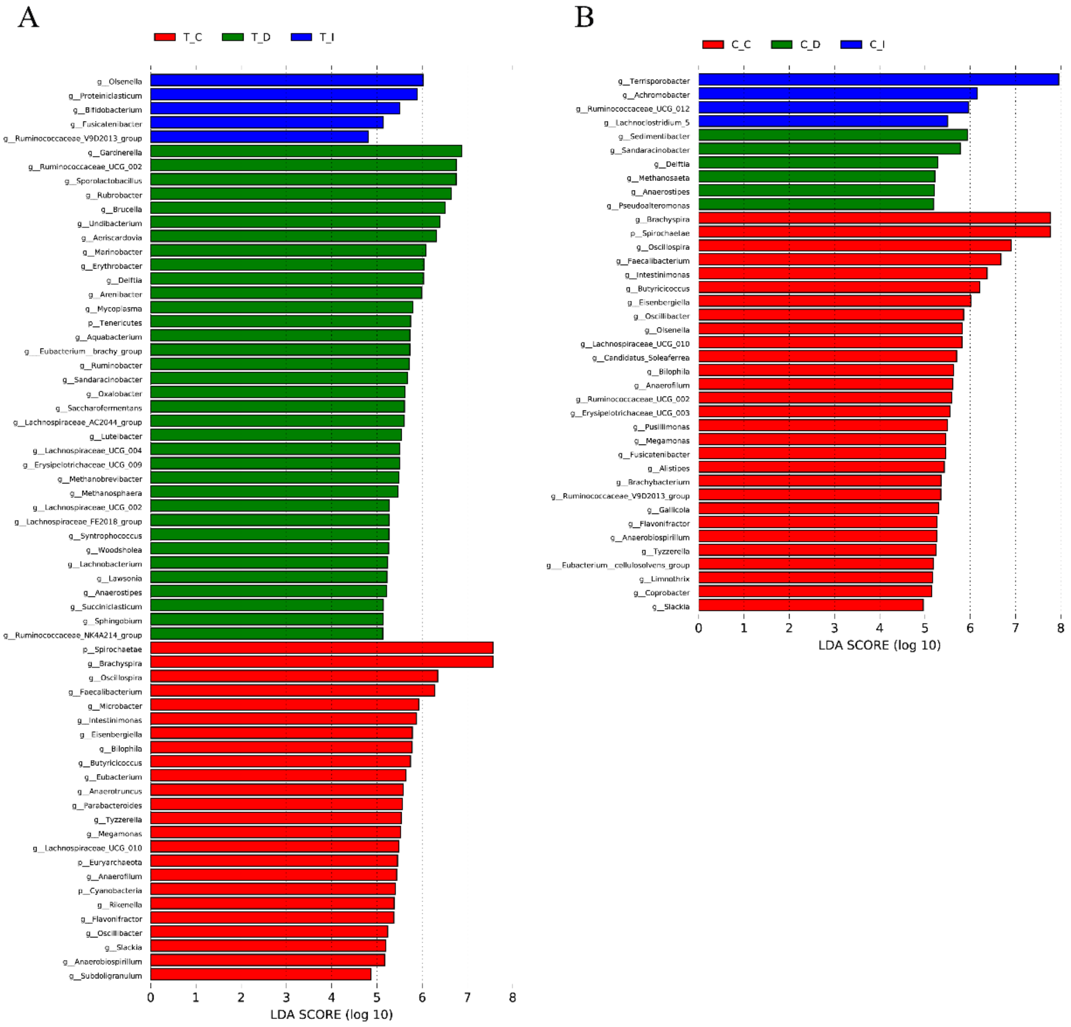

Supplementary Figure 3 Histogram of the Liner discriminant analysis (LDA) coupled with effects size measurements identified the most differentially abundant KEGG functional pathways of the 3 gut regions under the two rearing conditions. (A) The significant differential KEGG pathways of the 3 gut regions of the RPMF ducks. (B) The significant differential KEGG pathways of the 3 gut regions of the RLF ducks.

A

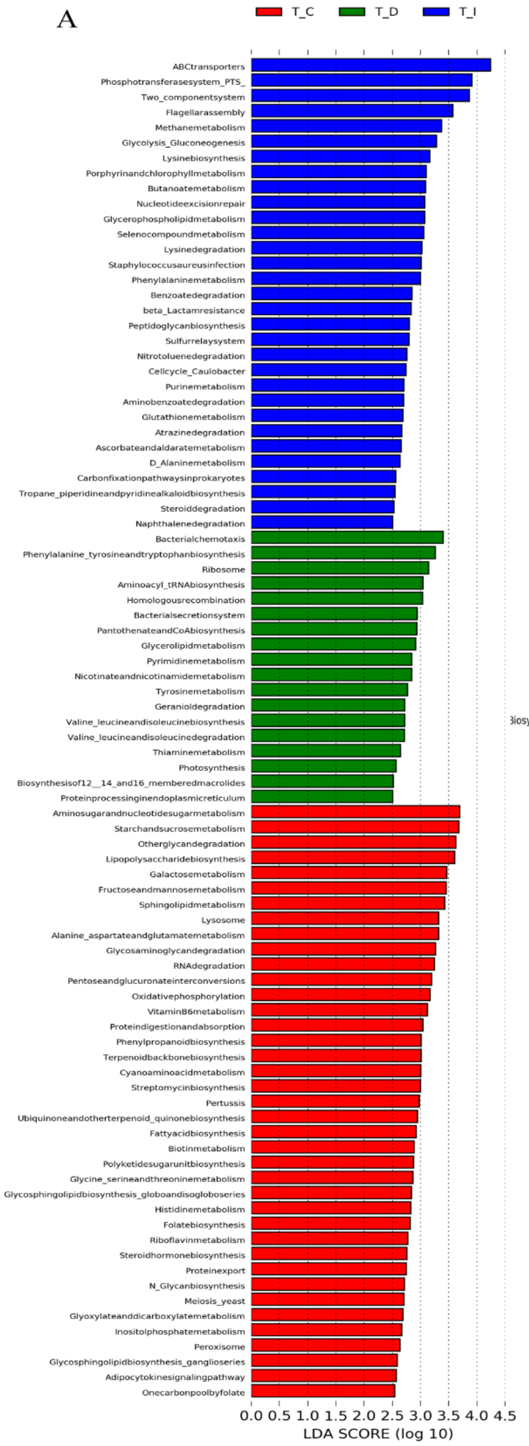

B

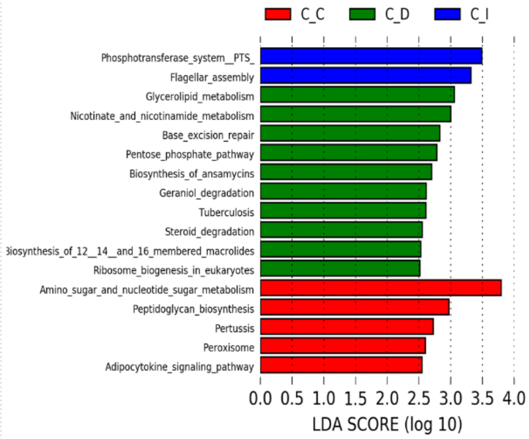

Supplementary Figure 4 Representative images of H&E-stained gut sections and liver tissues. (A-C) Intestine tissue of duodenum, ileum and cecum from RLF ducks (scale bar 150 μm). (D-F) Intestine tissue of duodenum, ileum and cecum from RPMF ducks (scale bar 150 μm). (G-H) Liver tissues from RLF ducks and RPMF ducks, respectively. The arrows showed specifics of inflammatory cell infiltrates (scale bar 150 μm).

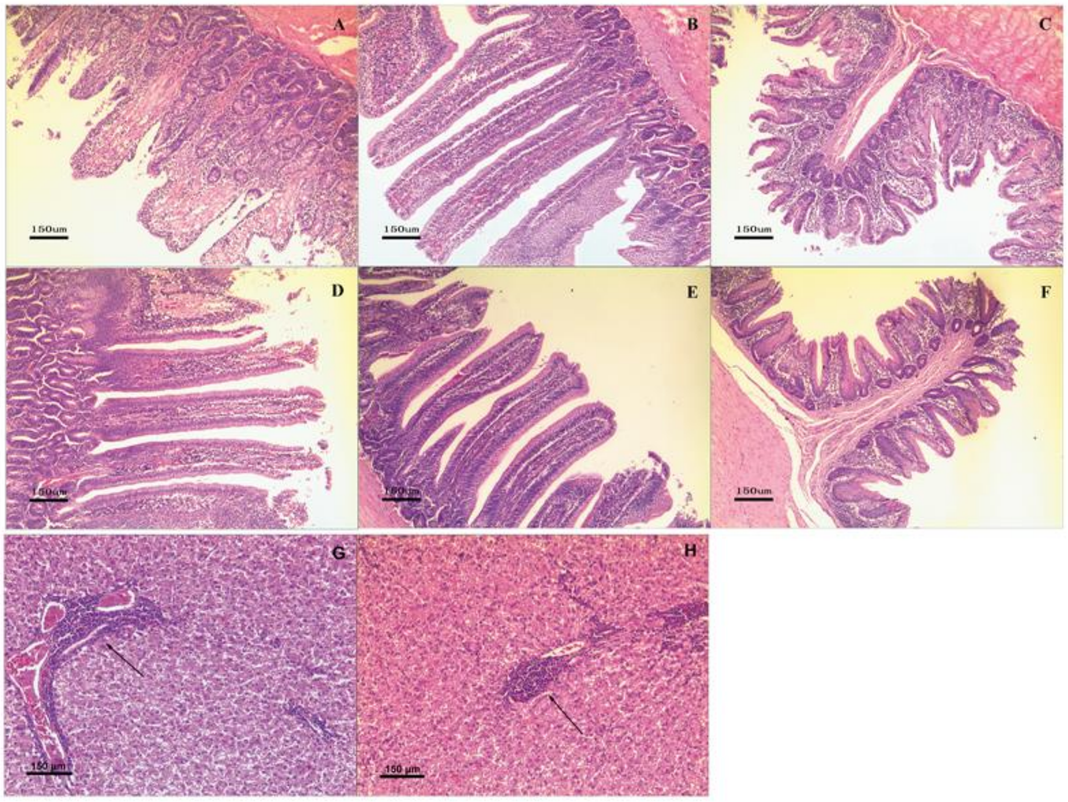

Supplementary Table 1 Influences of different rearing conditions on intestinal villi height (VH), crypts depth (CD), and mucosal thickness (MT) of different intestinal segments.

Intestinal segment	Item	Groups		P_value
		RPMF	RLF	
Duodenum	VH (μm)	761.2±116.67	639.2±225.34	0.04
	CD (μm)	298.1±99.01	368.6±85.80	0.021
	VH/CD	2.99±1.64	1.92±1.01	0.014
	MT (μm)	1043.63±150.00	961.78±135.58	0.449
Ileum	VH (μm)	862.0±216.95	918.7±202.66	0.665
	CD (μm)	173.27±36.01	278.66±58.12	1.00E-05
	VH/CD	5.11±1.53	3.50±1.20	1.00E-05
	MT (μm)	710.5±142.93	875.2±94.34	0.017
Cecum	CD (μm)	118.74±50.22	124.54±44.48	0.638
	MT (μm)	324.8±60.53	324.7±54.65	0.999

Supplementary Table 2 Influences of different rearing conditions on liver biochemical indexes.

Index	Groups		Reference range	P_value
	RPMF	RLF		
Superoxide dismutase / (U/mg)	9.28±1.68	10.43±1.17	40-120	0.005
total antioxidant capacity /(U/mg)	0.47±0.17	0.68±0.27	5-20	0.055
malondialdehyde / (nmol/mg)	0.38±0.09	0.32±0.05	2-10	0.048
Lipopolysaccharide /(EU/mg)	0.058±0.024	0.033±0.025	0.634	0.002
alanine transaminase /(U/g)	2.86±1.21	2.59±0.60	10-50	0.187
glutamic-oxalacetic transaminase /(U/g)	3.66±1.60	5.43±1.69	20-67	0.005
Lactic dehydrogenase/(U/g)	434.17±183.29	512.16±220.89	100-3000	0.118
Tumor necrosis factor-α/(pg/mg)	5.62±1.23	4.75±1.50	10-80	0.057

Supplementary Table 3 Influences of different rearing conditions on serum biochemical indexes.

Index	Groups		Reference range	P_value
	RPMF	RLF		
lysozyme/(U/ml)	249.83±141.00	293.90±123.98	100-500	0.291
IL-2/(pg/ml)	27.50±9.66	33.27±6.47	20-50	0.058
IL-4/(pg/ml)	10.02±2.72	7.66±1.98	42870	0.008
erepsin/（U/ml）	87.07±38.40	109.52±58.69	40-250	0.317
adrenaline/(pg/ml)	149.51±71.28	105.81±61.26	20-250	0.097
IgA/(g/L)	1.40±0.11	1.42±0.10	1-1.8	0.474
IgG/(g/L)	2.65±0.47	2.52±0.14	2-3.1	0.598
IgM/(g/L)	1.05±0.14	1.06±0.18	0.7-1.4	0.926
albumin/(g/L)	14.20±1.78	14.11±2.01	9-19	0.899
total protein/(g/L)	27.25±4.99	25.64±3.05	13-38	0.28
glutamic-oxalacetic transaminase/（U/L）	45.07±14.87	50.92±17.10	20-67	0.31
alanine transaminase/（U/L）	19.84±6.71	27.26±16.00	10-50	0.102
total antioxidant capacity/（U/ml）	15.52±2.11	14.45±2.03	5-20	0.156
prolactin/(uIU/ml)	27.09±15.57	25.61±10.67	10-50	0.777
estradiol/(pg/ml)	101.99±56.08	50.96±22.59	20-200	0.003
Follicle stimulating hormone/(mIU/ml)	3.07±1.36	2.81±1.09	0-20	0.651
Luteinizing hormone/(mIU/ml)	12.30±12.18	10.45±6.61	5-50	0.692
progesterone/(ng/ml)	1.14±0.85	0.82±0.41	0-5	0.191

Supplementary Table 4 The effect of different rearing conditions on duck body size traits.

Index	Groups		P_value
	RPMF	RLF	
weight（kg）	1.71±0.16	1.72±0.20	0.872
Body slanting length (cm)	22.74±1.22	22.59±1.68	0.775
keel length (cm)	11.40±0.98	11.12±0.70	0.359
Chest width (cm)	7.84±0.74	7.48±0.58	0.142
Chest depth (cm)	6.82±0.58	6.42±0.34	0.026
Pelvic width (cm)	6.33±0.47	6.23±0.43	0.545
Tibia length (cm)	5.17±0.37	4.97±0.38	0.136
shank girth (cm)	3.77±0.23	3.63±0.23	0.066
Semi-submersible length (cm)	50.67±1.31	47.90±11.61	0.955
